# Inhibition of fatty acid synthase protects obese mice from acute lung injury via ameliorating lung endothelial dysfunction

**DOI:** 10.1186/s12931-023-02382-w

**Published:** 2023-03-15

**Authors:** Zhuhua Wu, Li Zhu, Xinran Nie, Yingli Liu, Xiaoju Zhang, Yong Qi

**Affiliations:** 1 Department of Pulmonary and Critical Care Medicine, Henan Provincial People’s Hospital, Zhengzhou University People’s Hospital, No. 7, Weiwu Road, Zhengzhou, Henan China; 2 Department of Pulmonary and Critical Care Medicine, Zhengzhou University People’s Hospital, Henan Provincial People’s Hospital, Zhengzhou, Henan China

**Keywords:** Obesity, Fatty acid synthase, Acute lung injury, Endothelial cells, VE-cadherin

## Abstract

**Background:**

Obesity has been identified as a risk factor for acute lung injury/acute respiratory distress syndrome (ALI/ARDS). However, the underlying mechanisms remain elusive. This study aimed to investigate the role of fatty acid synthase (FASN) in lipopolysaccharide (LPS)-induced ALI under obesity.

**Methods:**

A high-fat diet-induced obese (DIO) mouse model was established and lean mice fed with regular chow diet were served as controls. LPS was intratracheally instilled to reproduce ALI in mice. In vitro, primary mouse lung endothelial cells (MLECs), treated by palmitic acid (PA) or co-cultured with 3T3-L1 adipocytes, were exposed to LPS. Chemical inhibitor C75 or shRNA targeting FASN was used for in vivo and in vitro loss-of-function studies for FASN.

**Results:**

After LPS instillation, the protein levels of FASN in freshly isolated lung endothelial cells from DIO mice were significantly higher than those from lean mice. MLECs undergoing metabolic stress exhibited increased levels of FASN, decreased levels of VE-cadherin with increased p38 MAPK phosphorylation and NLRP3 expression, mitochondrial dysfunction, and impaired endothelial barrier compared with the control MLECs when exposed to LPS. However, these effects were attenuated by FASN inhibition with C75 or corresponding shRNA. In vivo, LPS-induced ALI, C75 pretreatment remarkably alleviated LPS-induced overproduction of lung inflammatory cytokines TNF-α, IL-6, and IL-1β, and lung vascular hyperpermeability in DIO mice as evidenced by increased VE-cadherin expression in lung endothelial cells and decreased lung vascular leakage.

**Conclusions:**

Taken together, FASN inhibition alleviated the exacerbation of LPS-induced lung injury under obesity via rescuing lung endothelial dysfunction. Therefore, targeting FASN may be a potential therapeutic target for ameliorating LPS-induced ALI in obese individuals.

## Introduction

The prevalence rates of obesity have tripled over the past four decades, leading to adverse effects on public health [1]. Obesity predisposes to acute lung injury/acute respiratory distress syndrome (ALI/ARDS) [2]. A previous study demonstrated that obesity exhibited contrary immune responses to ALI in the setting of milder or more severe infection [3]. Other studies found that obesity protected mice from ventilator-induced lung injury [4]. Two recent studies indicated that obesity induced a proinflammatory phenotype and aggravated lung ischemia–reperfusion injury via regulating T-regulatory cell function [5, 6]. The influence of obesity on the pathological process of ALI/ARDS remains inconclusive. Endothelial cells, possessing physiological, immunological, and metabolic functions, establish a semi‐selective diffusion barrier to regulate the leakage of both inflammatory cells and fluid [7]. Endothelial dysfunction occurs early in ALI and plays a prominent role in the development of ALI [8]. Obesity is strongly associated with endothelial dysfunction due to oxidative stress, proinflammatory signaling pathways, and adipocyte-derived factors [9, 10]. However, little information is available regarding the effects of obesity on pulmonary microvascular endothelial function during LPS-induced ALI.

Fatty acid synthase (FASN) is a homodimeric, multifunctional polypeptide catalyzing palmitate production from acetyl-coenzyme A (CoA), malonyl-CoA, and NADPH that carry out de novo lipogenesis [11]. FASN is identified as a candidate gene for determining body fat [12]. Elevated lipogenesis has been associated with the pathogenesis of metabolic diseases, including obesity, type 2 diabetes, and nonalcoholic fatty liver disease [13–15]. The downregulation of FASN in type II alveolar epithelial cells contributes to ALI in diet-induced obese mice under hyperoxic exposure [16]. Previous studies demonstrated that FASN modulated endothelial dysfunction in hypoxic pulmonary artery endothelial cells [17, 18]. Overexpressed miR-335-5p was involved in alleviating the inflammatory response by targeting FASN in septic mouse models [19]. Recent studies have suggested a role for FASN in microvascular dysfunction in diabetes mellitus [20]. However, the role of FASN in lung endothelial cells in modulating LPS-induced ALI under obesity remains to be deciphered.

In this present study, we set out to identify the role of endothelial FASN in LPS-induced ALI in mice with obesity. We also performed a series of in vitro experiments to explore the role of FASN in modulating the endothelial mitochondrial function, endothelial adherens junction protein VE-cadherin, endothelial inflammation, and endothelial barrier function in lung endothelial cells under metabolic stress in response to LPS.

## Materials and methods

### Animals

C57BL/6 mice (6-week-old and 8-week-old, male) were purchased from Beijing Vital River Laboratory Animal Technology Co. Ltd. The mice were housed under conditions of the temperature of 22 ± 1 °C, the humidity of 45–55%, and a 12 h day/night circle. The animal experiments in this study were conducted per the guideline for the care and use of experimental animals. The Animal Ethics Committee of Henan Provincial Institute for Food and Drug Control approved the experiments involving animals.

### LPS-induced ALI murine model and C75 treatment

Six-week-old mice were randomly divided into a regular chow diet (Beijing Keao Xieli Feed Co., Ltd, 12% kcal fat) group (lean) or a high-fat diet (Research Diets, D12492, 60% kcal fat) group [diet-induced obese (DIO)]. After feeding for 24 weeks, the mouse model of ALI was established according to the previous literature [21]. Briefly, mice were anesthetized with pentobarbital and LPS (O55:B5, L2880, Sigma, 100 μg dissolved in 50 μL of saline) or vehicle (50 μL of saline) was instilled intratracheally into both lean and DIO mice. For FASN inhibition, DIO mice were intraperitoneally injected with C75 [MedChemExpress, HY-12364, dissolved in 10% dimethyl sulfoxide (DMSO)] at 10 mg/kg body weight at 30 min before LPS administration, and mice in other group were intraperitoneally injected with DMSO (vehicle) at the same concentration. Mice were euthanized and sacrificed at 6 h or 8 h after LPS administration, and their lung tissues were harvested for subsequent procedures.

### Isolation of lung endothelial cells

The lung endothelial cells were isolated from whole lung tissues using CD45 (Miltenyi Biotec, 130-052-301) and CD31 Microbeads (Miltenyi Biotec, 130-097-418) as previously described [22]. In brief, the harvested lungs were minced and digested with collagenase II (Gibco, 17101015) and filtered through a 100-μm nylon mesh. The cell suspension was resuspended in bovine serum albumin and incubated with anti-CD45-conjugated magnetic beads for negative selection, followed by positive selection with anti-CD31-conjugated magnetic beads. The CD31-positive cells were collected with an MACS separator (Miltenyi Biotec) following the manufacturer’s protocols. The purity of the endothelial cell population was confirmed by immunofluorescence of anti-CD31 (Abcam, ab7388) and anti-VE-cadherin (Affinity, AF6265). The primary mouse lung endothelial cells (MLECs) from 8-week-old male mice were cultured for in vitro experiments. Isolated MLECs were cultured with Dulbecco’s modified Eagle’s medium (DMEM) containing endothelial growth supplement, and the cells between passages 2 and 6 were used for in vitro experiments. The freshly isolated lung endothelial cells from lean and DIO mice were collected immediately to detect the protein levels by western blot.

### Palmitic acid treatment

For palmitic acid (PA, Sigma) treatment, PA combined with bovine serum albumin (BSA, Sigma) was added to the culture medium of MLECs. Briefly, sodium salt PA was dissolved in ethanol at 65 °C for 15 min and then allowed to combine with fatty acid free-BSA at final concentrations of 0.3 mM. After 24 h of PA treatment, MLECs were stimulated with 1 μg/mL LPS for 12 h and C75 (25 μM, 50 μM) was added 2 h prior to LPS. The solvent of ethanol with BSA and DMSO at the same concentration as the PA and C75, respectively, was used to treat control cells.

### Differentiation of 3T3-L1 cells into adipocytes and Transwell co-culture system

The 3T3‐L1 cells (iCell Bioscience Inc, iCell-m066) were cultured in DMEM supplemented with 10% FBS, 50 units/mL penicillin, and 50 μg/mL streptomycin in a humidified atmosphere of 5% CO_2_. The 3T3‐L1 cells were cultured for 2 days to confluence, and adipogenic differentiation was induced by treatment with DMEM containing 10% FBS, 0.5 mM IBMX, 5 µg/mL insulin, and 1 µM dexamethasone for 2 days. After differentiation induction, the medium was replaced with a differentiation–maintenance medium containing DMEM supplemented with 10% FBS and 5 µg/mL insulin and cultured for 4 days. Then, the cells were cultured in DMEM supplemented with 10% FBS, and the medium was replaced every 2 days. The differentiation efficiency of 3T3-L1 cells to adipocytes were assessed by Oil Red O staining. After differentiation into mature adipocytes, the medium was changed to the conditioned medium as performed with MLECs.

MLECs and mature adipocytes were co-cultured in a six-well Transwell system (Corning Inc., 3450) with a 0.4-μm porous membrane to separate the upper and lower chambers. The adipocytes were cultured in the lower chamber, while the MLECs were seeded and cultured in the upper chamber. After co-culture for 48 h, MLECs were removed from the upper chamber to a new culture chamber. Then, 1 μg/mL LPS was added into the medium and incubated for 12 h. C75 (25 μM, 50 μM) was added 2 h prior to LPS treatment. MLECs cultured without LPS or with DMSO at the same concentration as the C75 solvent were used as controls.

### shRNA-induced FASN gene silencing

For silencing of FASN, the MLECs were transfected with shRNA using the Lipofectamine 3000 Transfection Reagent (Invitrogen, USA) according to the manufacturer’s instructions. The sequences of FASN shRNA were as follows: sense, 5′-CTTTCTTCTTCGACTTCAAAG-3′, antisense, 5′-CTTTGAAGTCGAAGAAGAAAG-3′; the lentiviral control vector contained a non-sense FASN sequence [negative control (NC) shRNA]: sense, 5′-TTCTCCGAACGTGTCACGT-3′, antisense, 5′-ACGTGACACGTTCGGAGAA-3′. At 48 h post-transfection, validation of FASN knockdown was performed at the mRNA level using relative quantitative real-time polymerase chain reaction (qPCR) and at the protein level using western blot. After 24 h of PA treatment or 48 h of co-culture with adipocytes, the MLECs with FASN shRNA or NC shRNA were challenged by 1 μg/mL LPS for another 12 h.

### FASN activity assay

The lung homogenates and sonicated MLECs were centrifuged at 12,000×*g* for 20 min at 4 °C to obtain particle-free supernatants. FASN activity was estimated by a commercially available kit (Solarbio, BC0550), according to the manufacturer’s protocol. FASN activity was determined spectrophotometrically by measuring the decrease of absorption at 340 nm due to the oxidation of NADPH.

### Transmission electron microscopy

For mitochondrial ultrastructure observations, the treated MLECs were fixed overnight at 4 °C in 2.5% glutaraldehyde. The cells were rinsed in 0.1 M phosphate buffer (PH 7.4), and then fixed for 2 h in 1% osmic acid in buffer. Then the fixed cell mass were dehydrated in gradient ethanol solutions for dehydration, embedded into 812 embedding agent and polymerized at 60 °C for 48 h. Ultrathin sections of 70 nm were cut and post-stained with uranyl acetate and lead citrate. Mitochondrial morphology was observed under a transmission electron microscope (H7650, Hitachi, Japan).

### Analysis of mitochondrial membrane potential

The mitochondrial membrane potential (MMP) of MLECs was determined using the JC-1 fluorescent dye (Beyotime, C2006). MLECs were washed with ice‑cold phosphate-buffered saline (PBS) and incubated with JC-1 staining working solution at 37 °C for 20 min in the dark. Subsequently, the cells were washed twice with PBS, and the fluorescence intensity of the JC-1 polymer and JC-1 monomer was analyzed by a Novocyte flow cytometer. The mitochondrial membrane potential was shown as a ratio of the fluorescence intensity of polymers to monomers.

### Mitochondrial ROS detection

The mitochondrial reactive oxygen species (mtROS) contents were measured using MitoSOX (Maokang Biotech, M36008), a mitochondrial superoxide indicator. MLECs were washed with PBS three times and incubated with 5 μM MitoSOX at 37 °C for 15 min. After washing three times with PBS, the nuclei were stained with DAPI (1 μM) for 15 min. The photographs were captured using a microscope (BX53, Olympus, Japan) and the red fluorescence intensity was measured. The measurement was also performed using a Novocyte flow cytometer and analyzed using the FlowJo software.

### Western blot analysis

The lung tissues/cells were homogenized in RIPA buffer containing protease and phosphatase inhibitors. The lysates were centrifuged at 14,000 rpm (15 min, 4 °C), and the supernatant was collected for further analysis. The proteins were separated with SDS-polyacrylamide gels and then transferred to a polyvinyl difluoride (PVDF) membrane (Millipore, IPVH00010). Immunoblotting was performed at 4 °C overnight using primary antibodies directed against FASN (1:2000, Abcam, ab22759), p38 MAPK (1:500, Proteintech, 14064-1-AP), phospho (p)-p38 MAPK (Thr180/Tyr182) (1:1000, Proteintech, 28796-1-AP), NLRP3 (1:500, wanleibio, WL02635), VE-cadherin (1:1000, Affinity, AF6265), Drp1 (1:2000, Proteintech, 12957-1-AP), phospho (p)-Drp1 (Ser616) (1:500, Affinity, AF8470), Mfn2 (1:1000, abclonal, A19678) and β-actin (1:2000, Proteintech, 60,008–1-Ig). The membranes were then incubated with the secondary antibody (1:10,000, SA00001-1, SA00001-2, Proteintech) at room temperature for 1 h. The protein bands were detected using ECL solution (E003, 7 Sea Biotech, China) and visualized using the ImageJ software (v.1.53c; NIH).

### Quantitative real-time PCR

Total RNA was isolated from lung tissues or MLECs using the TRIzol Reagent (Invitrogen). For the quantitative real-time PCR (qPCR), cDNA was synthesized using PrimeScript RT Master Mix (TaKaRa, Japan) following the manufacturer’s protocols. qPCR was performed on cDNA using TB Green Premix Ex Taq II (Tli RNaseH Plus) (TaKaRa) and ROX Reference Dye (TaKaRa) on a ABI 7900HT Real-Time PCR System (Thermo Fisher Scientific Inc.). The mRNA levels of interleukin-6 (IL-6), tumor necrosis factor-alpha (TNF-α), and interleukin-1β (IL-1β) were calculated using the comparative threshold method (ΔΔCt), and normalized to endogenous control β-actin. The primers and probes were as follows:

- mouse IL-6 forward, 5′-ATGGCAATTCTGATTGTATG-3′;
- mouse IL-6 reverse, 5′-GACTCTGGCTTTGTCTTTCT-3′;
- mouse TNF-α forward, 5′-CAGGCGGTGCCTATGTCTCA-3′;
- mouse TNF-α reverse, 5′-GCTCCTCCACTTGGTGGTTT-3′;
- mouse IL-1β forward, 5′-CTCAACTGTGAAATGCCACC-3′;
- mouse IL-1β reverse, 5′-GAGTGATACTGCCTGCCTGA-3′;
- mouse β-actin forward, 5′-GGCACCCAGCACAATGAA-3′;
- mouse β-actin reverse, 5′-TAGAAGCATTTGCGGTGG-3′.

### Immunofluorescence staining

For immunofluorescence assay, MLECs were grown on coverslips in 24-well plates. The cells were fixed with 4% paraformaldehyde and permeabilized with 0.5% Triton X-100 in PBS. After blocking with serum blocking solution, the cells were incubated with rabbit antibody to VE-cadherin (Affinity, AF6265, 1:200) overnight at 4 °C and then incubated with secondary antibody fluorescein isothiocyanate (FITC)-labeled goat anti-rabbit IgG (Abcam, ab6717, 1:200) for 1 h at room temperature followed by DAPI staining (Aladdin reagent, D106471). Representative images were further captured under a microscope (BX53, Olympus, Japan).

### Measurements of endothelial cell permeability

For permeability studies, MLECs were grown on the apical side of the collagen-coated polyester membrane of a Transwell (0.4 μm pore size). After 24 h of PA treatment or 48 h of co-culture with adipocytes, the MLEC monolayer was treated with or without LPS (1 μg/mL, 12 h) in the presence or absence of C75 (25 μM, 50 μM). C75 was added 2 h prior to the addition of LPS. The solvent controls were added as above. Then, 1 mg/mL fluorescein isothiocyanate (FITC)-dextran was administered in the upper chamber. The fluorescence intensity of the medium from each chamber was measured using BioTek Synergy H1 (Biotek) 30 min after the stimulation of dextran. The flux was expressed as the percentage of dextran diffusion per hour per square centimeter [23].

### Transendothelial electrical resistance

The cellular barrier properties were assessed by measuring the transendothelial electrical resistance (TEER) across MLECs using Millicell-ERS (MERS00002, Millipore). MLECs were grown to a confluent monolayer on the apical side of the polycarbonate membrane of a Transwell (0.4 μm pore size). After 24 h of PA treatment or 48 h of co-culture with adipocytes, the MLEC monolayer was treated with or without LPS (1 μg/mL, 12 h) in the presence or absence of C75 (25 μM, 50 μM). C75 was added 2 h prior to the addition of LPS. The solvent controls were added as above. The TEER across MLEC monolayer was measured with the electrode tips in the upper and lower compartments at various time points and expressed as Ω cm^2^ [24].

### Enzyme-linked immunosorbent assay

The levels of TNF-α, IL-6, and IL-1β in mouse lung homogenates and cell culture supernatant were assayed using commercial ELISA kits following the manufacturer’s protocols (Cloud-Clone Corp, China).

### Lung histology and lung injury score

The left upper lung lobes were collected from mice 8 h after LPS instillation and they were fixed with 4% paraformaldehyde for 24 h. The tissue sections were embedded in paraffin and cut into 5-μm-thick sections. The tissue sections were stained with hematoxylin and eosin (H&E), and the lung injury was evaluated based on the hemorrhage, hyperemia, edema of the alveolar wall, and inflammatory cell infiltration according to the previously reported protocol [25].

### Lung vascular leak assessment

The lung vascular leak was measured in each experimental group using (1) Evans blue dye (EBD) extravasation assay in lung tissues, (2) lung wet-to-dry ratio measurement, and (3) total protein concentrations in bronchoalveolar lavage fluid (BALF). After 8 h of LPS instillation, EBD (20 mg/kg; Macklin, E6135) were injected into the mice via the tail vein and allowed to circulate in the blood vessels for 3 h. The anticoagulant blood was collected and centrifuged at 3000 rpm for 20 min. Then, the intravascular EBD in the lung was washed through the right ventricle with saline for 5 min. EBD was extracted from the lungs by incubation with formamide (60 °C for 24 h) and then centrifuged at 5000 rpm for 30 min. The absorbance of plasma and lung supernatants was measured by spectrophotometry at 620 nm and corrected for the presence of heme pigments as follows: *A*_620_ (corrected) = *A*_620_ − (1.426 × *A*_740_ + 0.030) [26]. Evans blue leaking index was calculated as the ratio of the dye concentration in the lung to the plasma.

The right lung lobes were collected and weighed immediately (wet weight) at 8 h following LPS instillation. The samples were reweighed after dried in a 60 °C oven for 48 h (dry weight). The ratio of wet weight to dry weight (W/D) was calculated.

BALF was collected by cannulating the trachea with a blunt 22-gauge needle and then lavaging the lungs four times with 0.75 mL of ice-cold PBS. The levels of total protein content in BALF was determined using a bicinchoninic acid protein assay kit (Solarbio, PC0020).

### Statistical analysis

The values were expressed as the mean ± standard deviation (SD). Unpaired-sample Student *t* tests were used to analyze the differences between the two groups when appropriate. Multi-group comparisons were performed using two-tailed ANOVA with the post hoc analysis of least significant difference (LSD) or Games-Howell method. For all analyses, the two-tailed *P* < 0.05 indicated a statistically significant difference. SPSS version 26.0 was used for statistical analysis.

## Results

### FASN expression in lung endothelial cells increased under obesity during LPS-induced ALI

**Fig. 1:**
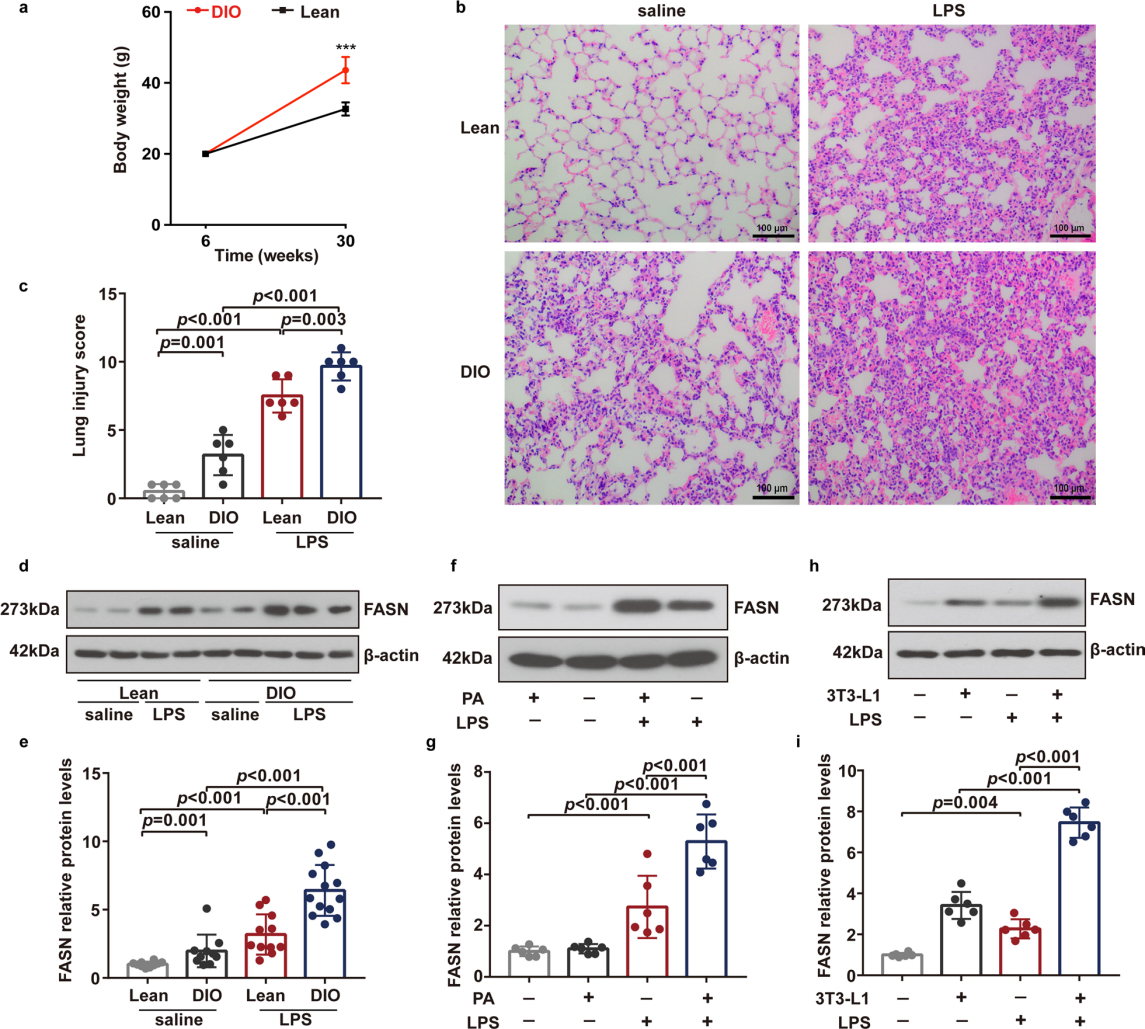
Expression levels of FASN were upregulated in lung endothelial cells under obesity or metabolic stress in response to LPS. **a** Body weight of lean and DIO mice during regular chow diet and high-fat diet feeding, respectively; *n* = 83 in the lean mice group and *n* = 125 in the DIO mice group. Student’s *t* test analysis. **b**, **c** Representative H&E images of the lungs (**b**) and quantitative analysis of the lung injury (**c**) in the lean and DIO mice 8 h after LPS or saline intratracheal administration; *n* = 6 mice in each group. Scale bar: 100 μm. One-way ANOVA and LSD posthoc analysis. **d**, **e** Representative western blots (**d**) and quantitative analysis (**e**) of FASN expression in freshly isolated lung endothelial cells from the lean and DIO mice 6 h after LPS or saline intratracheal administration; *n* = 10–13 in each group. One-way ANOVA and LSD posthoc analysis. **f**–**i** Representative western blots (**f**, **h**) and quantitative analysis (**g**, **i**) of FASN expression in each group of mouse lung endothelial cells (MLECs). *n* = 6 biologically independent experiments. One-way ANOVA and LSD or Games–Howell posthoc analysis. Data were represented as mean ± SD

High-fat diet-fed mice (obese group) showed significantly higher body weight gains than regular chow diet-fed mice (lean group) (Fig. 1a). The histology scores of lung injury by LPS intratracheal instillation (Fig. 1b, c) were significantly higher in the lung tissues of obese mice than those in lean mice, indicating that obesity exhibited the aggravation of LPS-induced ALI. Pulmonary endothelium is critically involved in the pathogenesis of ALI [8]. We examined the expression of FASN in freshly isolated lung endothelial cells to assess the involvement of FASN in obesity-induced ALI exacerbation. The expression level of FASN was significantly elevated in lung endothelial cells isolated from obese mice versus lean mice and further elevated following LPS challenge (Fig. 1d, e).

We cultured MLECs under metabolic stress including PA or co-culture with adipocytes, which were established as the in vitro models of obese conditions. Consistently, we observed that the co-culture with adipocytes significantly increased FASN expression in MLECs. Importantly, the expression levels of FASN were remarkably upregulated in MLECs under PA or co-culture with adipocytes compared with control MLECs following LPS stimulation, suggesting a key role for FASN in mediating the functional alterations of lung endothelial cells undergoing metabolic stress in response to LPS (Fig. 1f–i). We also observed that LPS resulted in increased FASN activity in MLECs, and PA induced further increase of FASN activity in MLECs as compared to control MLECs when exposed to LPS, which was inhibited by C75 treatment (Fig. 3k).

### Involvement of FASN in mitochondrial dysfunction in MLECs under metabolic stress when exposed to LPS

**Fig. 2:**
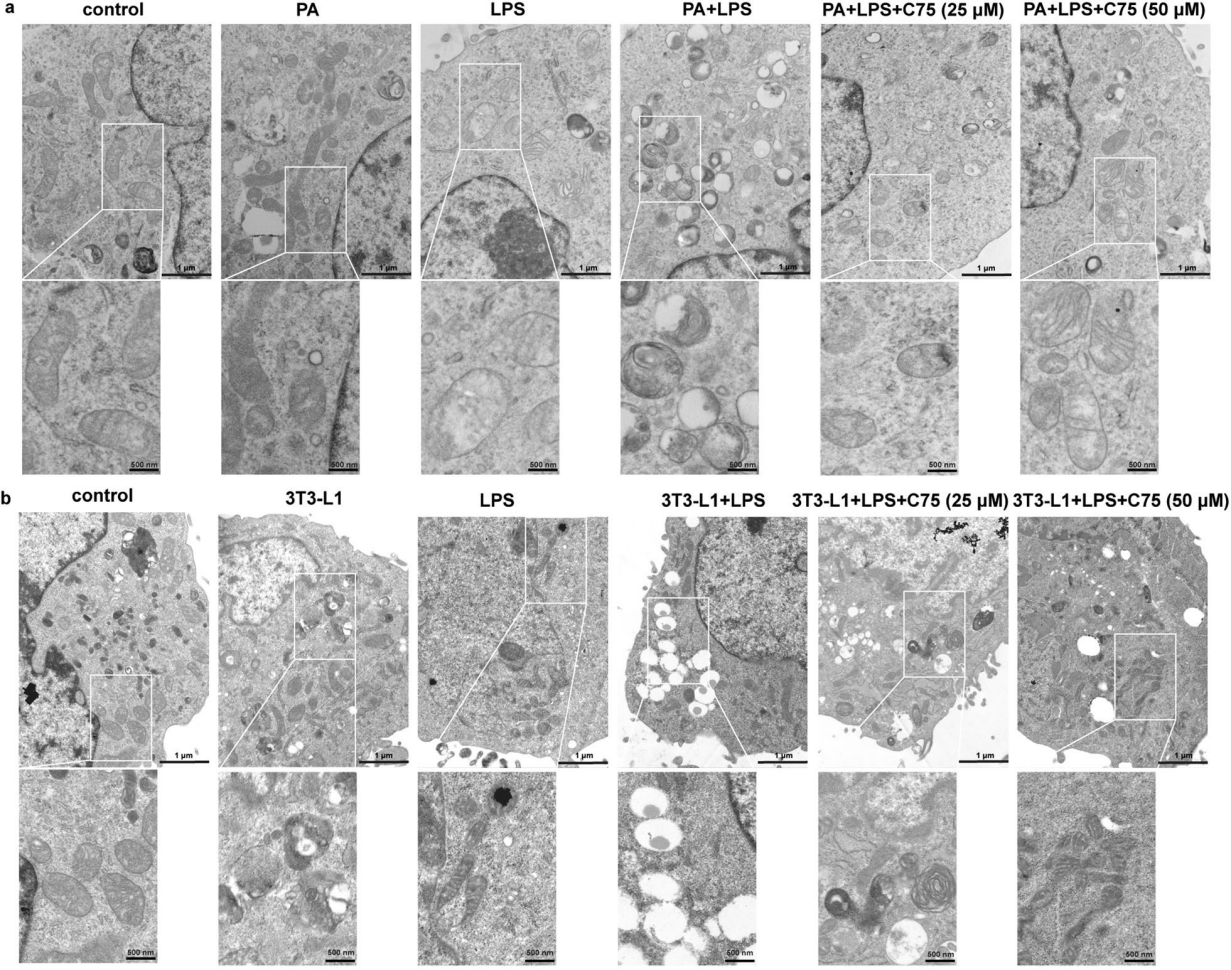
FASN regulated the mitochondrial dynamics in MLECs under metabolic stress in response to LPS. **a** Mitochondrial morphology was visualized by transmission electron microscopy in MLECs under PA in response to LPS with or without C75 treatment. **b** Mitochondrial morphology was visualized by transmission electron microscopy in MLECs co-cultured with 3T3-L1 adipocytes following LPS exposure with or without C75 treatment. Scale bar: 1 μm; 500 nm

**Fig. 3:**
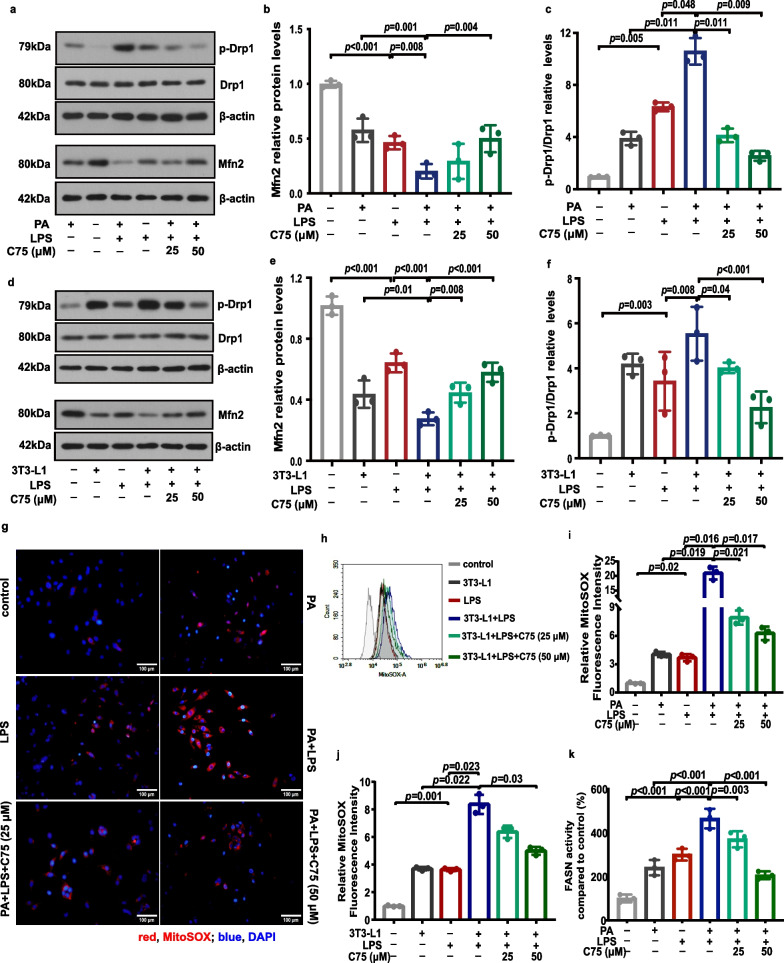
FASN regulated the levels of mitochondrial ROS in MLECs under metabolic stress in response to LPS. **a**–**f** Representative western blots of Mfn2, p-Drp1 and Drp1 (**a**, **d**), and corresponding quantitative analysis of Mfn2 (**b**, **e**) and p-Drp1/Drp (**c**, **f**) in each group of MLECs. *n* = 3 biologically independent experiments. One-way ANOVA and LSD or Games–Howell posthoc analysis. **g–j** Representative immunofluorescence images (**g**), flow cytometry analysis (**h**) and quantitative analysis (**i**, **j**) of mitoSOX in each group of MLECs. *n* = 3 biologically independent experiments. Scale bar: 100 μm. One-way ANOVA and Games–Howell posthoc analysis. **k** FASN activity were detected in MLECs under PA in response to LPS with or without C75 treatment. One-way ANOVA and LSD posthoc analysis. *n* = 3 biologically independent experiments. Data were represented as mean ± SD

**Fig. 4:**
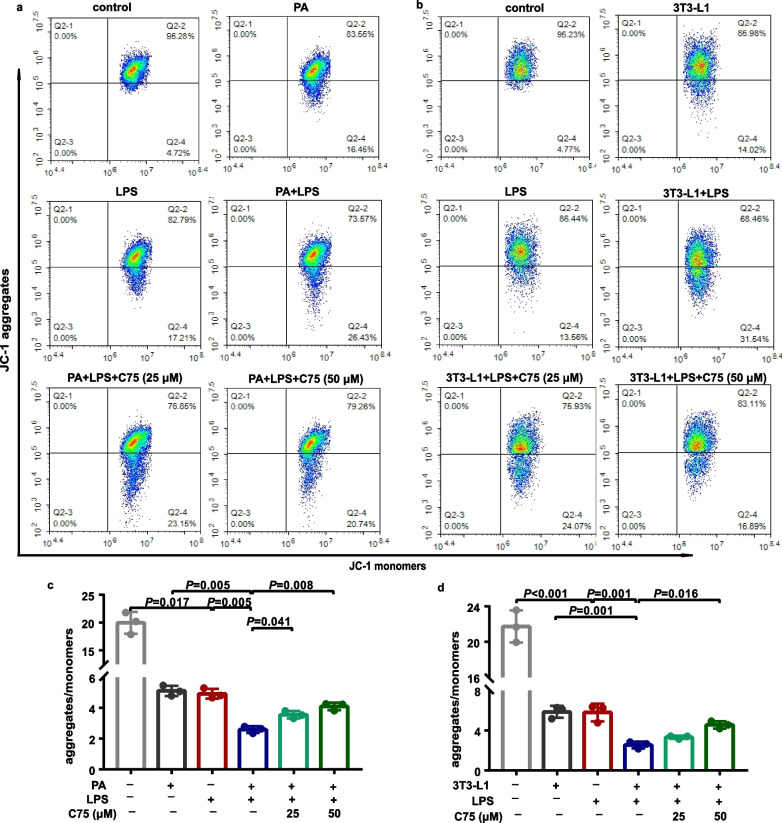
FASN regulated the mitochondrial membrane potential in MLECs under metabolic stress in response to LPS. Representative flow cytometry analysis (**a**, **b**) and quantitative analysis (**c**, **d**) of the mitochondrial membrane potential in each group of MLECs. *n* = 3 biologically independent experiments. One-way ANOVA and LSD or Games–Howell posthoc analysis. Data were represented as mean ± SD

First, we investigated the role of FASN in regulating mitochondrial function by determining the mtROS level and MMP in MLECs under metabolic stress. Ample evidence indicates that mitochondrial homeostasis is crucial for endothelial integrity and mitochondria are the main source of ROS production [27, 28]. We examined the mitochondrial ultrastructure using transmission electron microscopy. MLECs under metabolic stress induced by PA or adipocytes when exposed to LPS exhibited mitochondria ultrastructural changes, including irregular arrangement, vacuolation, and loss of cristae, which were ameliorated after C75 treatment (Fig. 2a, b). Moreover, the level of Drp1 phosphorylation was greater and the protein expression of Mfn2 were lower in PA- (Fig. 3a–c) or adipocyte-treated (Fig. 3d–f) MLECs as compared to control MLECs following LPS challenge, which were partially reversed in the C75 treatment group. We found that the levels of mtROS were significantly elevated in MLECs under PA or adipocytes treatment with or without the LPS challenge (Fig. 3g–j). Treatment with C75, an inhibitor of FASN, attenuated mtROS overproduction in MLECs under metabolic stress following LPS stimulation (Fig. 3g–j). Besides, we observed that the MMP of MLECs was remarkably decreased in the presence of PA or adipocytes and further decreased following LPS challenge (Fig. 4). As shown in Fig. 4, inhibition of FASN by C75 could partially restore LPS-induced mitochondrial membrane polarization as evidenced by the increased MMP in PA- or adipocyte-treated MLECs.

### FASN modulated p38 MAPK/NLRP3/VE-cadherin signaling in MLECs under metabolic stress when exposed to LPS

**Fig. 5:**
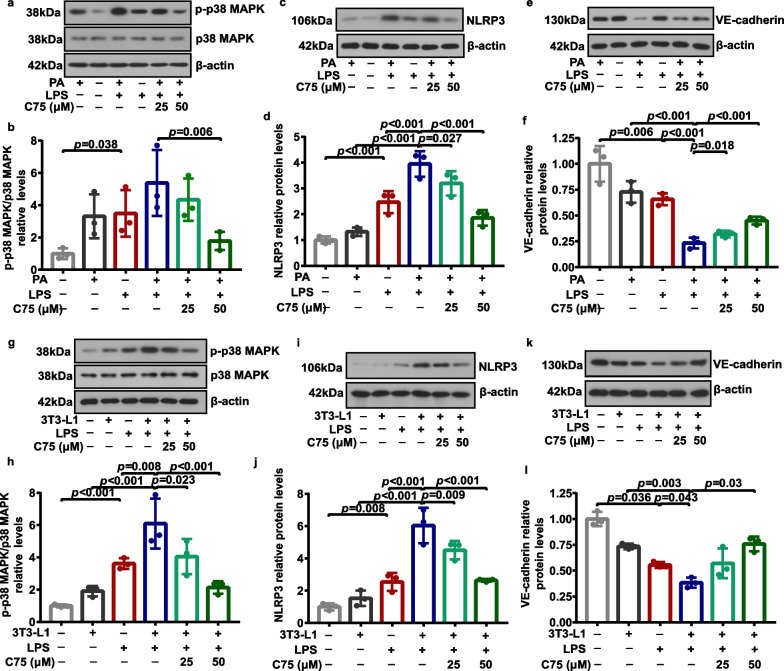
Involvement of FASN in regulating the p38 MAPK/NLRP3/VE-cadherin signaling in MLECs under metabolic stress when subjected to LPS. Representative western blots of p-p38 MAPK (**a**, **g**), p38 MAPK (**a**, **g**), NLRP3 (**c**, **i**), and VE-cadherin (**e**, **k**), and corresponding quantitative analysis of p-p38 MAPK/p38 MAPK (**b**, **h**), NLRP3 (**d**, **j**), and VE-cadherin (**f**, **l**) in each group of MLECs. *n* = 3 biologically independent experiments. One-way ANOVA and LSD or Games–Howell posthoc analysis. Data were represented as mean ± SD

**Fig. 6:**
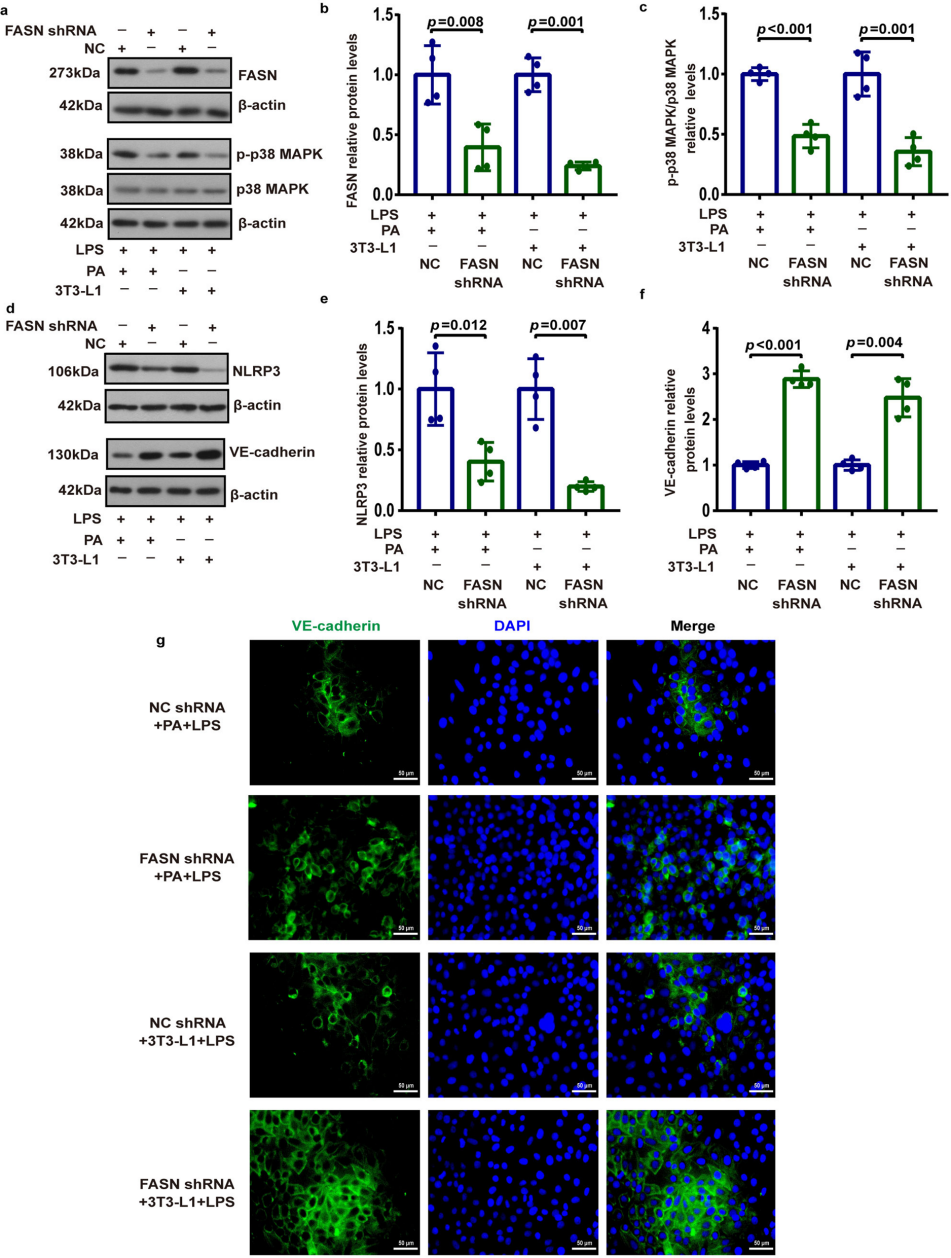
Deficiency of FASN suppressed the p38 MAPK/NLRP3 signaling and increased VE-cadherin expression levels in MLECs under metabolic stress when subjected to LPS. **a**–**f** Representative western blots of FASN (**a**), p-p38 MAPK (**a**), p38 MAPK (**a**), NLRP3 (**d**), and VE-cadherin (**d**), and quantitative analysis of FASN (**b**), p-p38 MAPK/p38 MAPK (**c**), NLRP3 (**e**), and VE-cadherin (**f**) expression in each group of MLECs. *n* = 4 biologically independent experiments. Student’s *t* test analysis. **g** Representative immunofluorescence images of VE-cadherin expression in each group of MLECs. Scale bar: 50 μm. Data were represented as mean ± SD

Pulmonary endothelial barrier dysfunction is the central pathophysiological feature of ALI, and the microvascular barrier function depends on the adherens junction proteins at the endothelial cell–cell borders [29]. We next attempted to explore the regulation of FASN on lung endothelial adherens junction protein VE-cadherin and the potential involved signaling pathways under metabolic stress in response to LPS. We observed that the protein levels of phosphorylated p38 MAPK (Fig. 5a, b, g, h) and NLRP3 (Fig. 5c, d, i, j) were elevated while the protein levels of VE-cadherin (Fig. 5e, f, k, l) were reduced in MLECs following LPS treatment. We identified that MLECs co-cultured with adipocytes showed remarkably higher protein levels of phosphorylated p38 MAPK than control MLECs following the LPS challenge (Fig. 5g, h). Besides, LPS administration predominantly induced a further increase in the expression of NLRP3 (Fig. 5c, d, i, j) and a further decrease in the expression of VE-cadherin (Fig. 5e, f, k, l) in MLECs after PA treatment or co-culture with adipocytes. As expected, the inhibition of FASN with C75 or corresponding shRNAs (Fig. 6a, b) increased the expression of VE-cadherin (Figs. 5e, f, k, l, 6d, f) in MLECs under metabolic stress following LPS administration, which was accompanied by decreases in the phosphorylation of p38 MAPK (Figs. 5a, b, g, h, 6a, c) and NLRP3 expression (Figs. 5c, d, i, j, 6d, e). Moreover, images of immunofluorescence staining showed that the expression of VE-cadherin was enhanced in MLECs with FASN silencing under metabolic stress following LPS challenge (Fig. 6g).

### Involvement of FASN in endothelial barrier dysfunction and endothelial inflammation in MLECs under metabolic stress when exposed to LPS

**Fig. 7:**
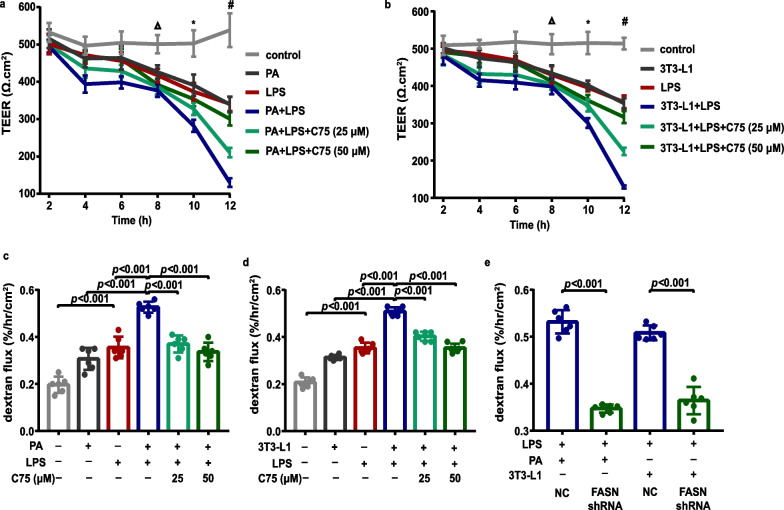
FASN regulated the endothelial barrier function in MLECs under metabolic stress when exposed to LPS. **a** The transendothelial electrical resistance was measured in PA-treated MLECs following LPS exposure with or without C75 treatment. ∆, *P* = 0.016, control group versus LPS group. *, *P* = 0.003, control group versus LPS group; *P* = 0.021, LPS group versus PA + LPS group. #, *P* = 0.001, control group versus LPS group; *P* < 0.001, LPS group versus PA + LPS group, PA + LPS group versus PA + LPS + C75 (25 μM, 50 μM) group. *n* = 3 biologically independent experiments. One-way ANOVA and LSD posthoc analysis. **b** The transendothelial electrical resistance in MLECs co-cultured with 3T3-L1 adipocytes following LPS exposure with or without C75 treatment. ∆, *P* = 0.016, control group versus LPS group. *, *P* < 0.001, control group versus LPS group; *P* = 0.002, LPS group versus 3T3-L1 + LPS group; *P* = 0.029, 3T3-L1 + LPS group versus 3T3-L1 + LPS + C75 group (50 μM) group. #, *P* < 0.001, control group versus LPS group, LPS group versus 3T3-L1 + LPS group, 3T3-L1 + LPS group versus 3T3-L1 + LPS + C75 (25 μM, 50 μM). *n* = 3 biologically independent experiments. One-way ANOVA and LSD posthoc analysis. **c** The MLEC monolayer permeability was detected by the dextran flux in PA-treated MLECs following LPS exposure with or without C75 treatment. *n* = 6 biologically independent experiments. One-way ANOVA and LSD posthoc analysis. **d** The MLEC monolayer permeability was detected by the dextran flux in MLECs co-cultured with 3T3-L1 adipocytes following LPS exposure with or without C75 treatment. *n* = 6 biologically independent experiments. One-way ANOVA and LSD posthoc analysis. **e** The MLEC monolayer permeability was detected by the dextran flux in MLECs with FASN shRNA or NC shRNA under PA or adipocytes treatment when exposed to LPS. *n* = 6 biologically independent experiments. Student’s *t* test analysis. Data were represented as mean ± SD

**Fig. 8:**
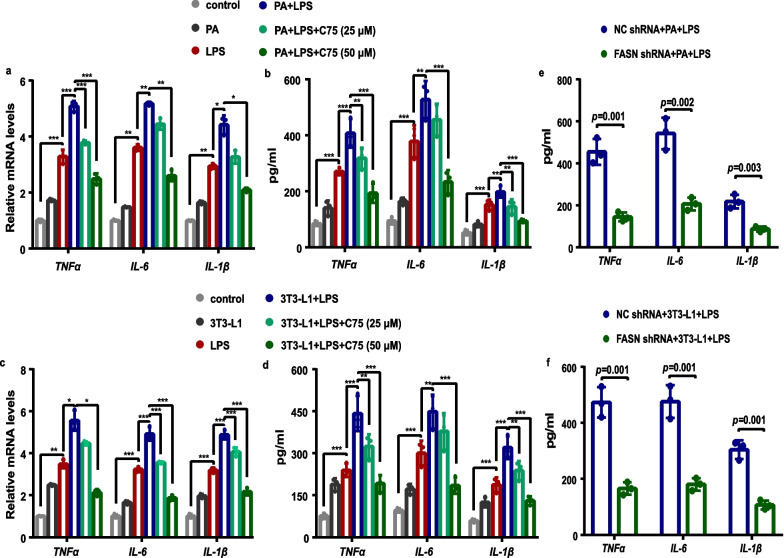
Inhibition of FASN decreased endothelial inflammation in MLECs under metabolic stress when exposed to LPS. **a**–**d** The mRNA level of TNF-α, IL-6, and IL-1β in each group of MLECs were measured by qPCR (**a**, **c**). The secretion levels of TNF-α, IL-6, and IL-1β in the cell supernatant were measured by ELISA assay in each group of MLECs (**b**, **d**). One-way ANOVA and LSD or Games–Howell posthoc analysis. **e**, **f** The secretion levels of TNF-α, IL-6, and IL-1β in the cell supernatant were measured by ELISA assay in MLECs with FASN shRNA or NC shRNA under PA (**e**) or adipocytes (**f**) treatment when exposed to LPS. Student’s *t* test analysis. *n* = 3 biologically independent experiments. Data were represented as mean ± SD

We next sought to examine the effect of FASN on endothelial barrier function in MLECs under metabolic stress. As shown in Fig. 7a, b, the levels of TEER across MLEC monolayers, recorded as an assessment of barrier integrity, exhibited a marked decline after 8 h of LPS stimulation. Compared with the levels in the LPS-stimulated MLECs, MLECs under PA treatment exhibited markedly lower TEER after 10 h of the LPS challenge (Fig. 7a), and MLECs co-cultured with adipocytes showed remarkably lower TEER after 10 h of the LPS challenge (Fig. 7b). The monolayer permeability of in vitro MLECs was calculated by the relative fluorescence of FITC-dextran, which exhibited an opposite trend to the TEER. Comparably, LPS stimulation significantly increased endothelial cell permeability (Fig. 7c, d), and the mRNA levels (Fig. 8a, c) and secretion levels (Fig. 8b, d) of inflammatory cytokines TNF-α, IL-6, and IL-1β in MLECs after 12 h of the LPS challenge. More importantly, PA or adipocytes-treated MLECs displayed markedly enhanced endothelial cell permeability (Fig. 7c, d), and increased levels of TNF-α, IL-6 and IL-1β (Fig. 8a–d) as compared to control MLECs in response to LPS. As expected, FASN inhibition with C75 or gene silencing with shRNAs targeting FASN attenuated these effects as evidenced by elevated TEER (Fig. 7a, b), decreased MLEC monolayer permeability (Fig. 7c–e), and decreased levels of inflammatory cytokines TNF-α, IL-6, and IL-1β (Fig. 8).

### FASN inhibition restored VE-cadherin expression in lung endothelial cells and protected against LPS-induced ALI in obese mice via suppressing pulmonary microvascular leakage

**Fig. 9:**
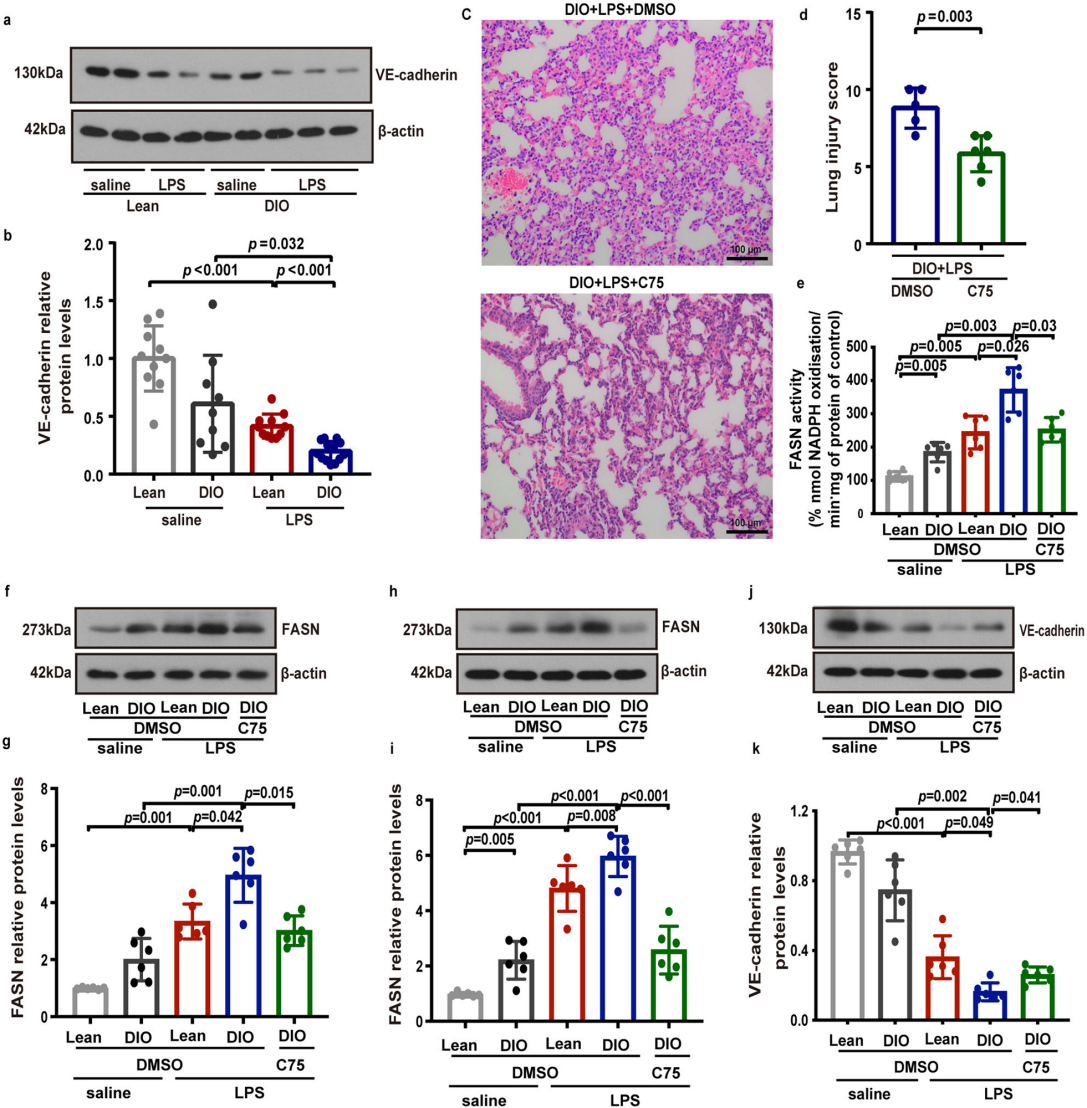
C75 significantly increased the expression levels of VE-cadherin in lung endothelial cells from DIO mice. **a**, **b** Representative western blots (**a**) and quantitative analysis (**b**) of VE-cadherin expression in freshly isolated lung endothelial cells from the lean and DIO mice at 6 h after LPS or saline intratracheal administration. *n* = 10–13 mice in each group. One-way ANOVA and Games–Howell posthoc analysis. **c**, **d** HE images of lungs (**c**) and assessment of the lung injury (**d**) in LPS-treated DIO mice with or without C75 pretreatment. *n* = 5–6 mice in each group. Scale bar: 100 μm. Student’s *t* test analysis. **e**–**k** The lean and DIO mice were intratracheally instilled LPS or saline for 8 h. C75 was intraperitoneally administered in DIO mice 30 min before LPS instillation. The FASN activity of lung tissues were detected in each group of mice (**e**). Representative western blots and quantitative analysis of FASN in lung tissues (**f**, **g**), and FASN (**h**, **i**) and VE-cadherin (**j**, **k**) in freshly isolated lung endothelial cells in each group of mice. *n* = 6 mice in each group. One-way ANOVA and LSD or Games–Howell posthoc analysis. Data were represented as mean ± SD

To further investigate the in vivo effect of FASN on lung endothelial function under obesity conditions, the protein levels of VE-cadherin were determined in freshly isolated lung endothelial cells from obese and lean mice. We found that the protein levels of VE-cadherin were remarkably reduced in lung endothelial cells from both obese and lean mice with LPS challenge (Fig. 9a, b). Moreover, as shown in Fig. 9a, b, obese mice exhibited a significant decrease in the endothelial expression of VE-cadherin compared with lean mice following LPS challenge. Compared to lean mice, we found that the FASN activity was increased in lung tissue from obese mice following LPS exposure, which was reversed by C75 treatment (Fig. 9e). We also found that the expression level of FASN in lung tissue were decreased in obese mice with LPS challenge after C75 treatment (Fig. 9f, g). C75 treatment predominantly suppressed FASN expression (Fig. 9h, i) and restored VE-cadherin expression (Fig. 9j, k) in lung endothelial cells from obese mice with LPS challenge. Consistently, the histological analysis showed an obvious amelioration in pathological lung injury in obese mice after treatment with C75 (Fig. 9c, d).

**Fig. 10:**
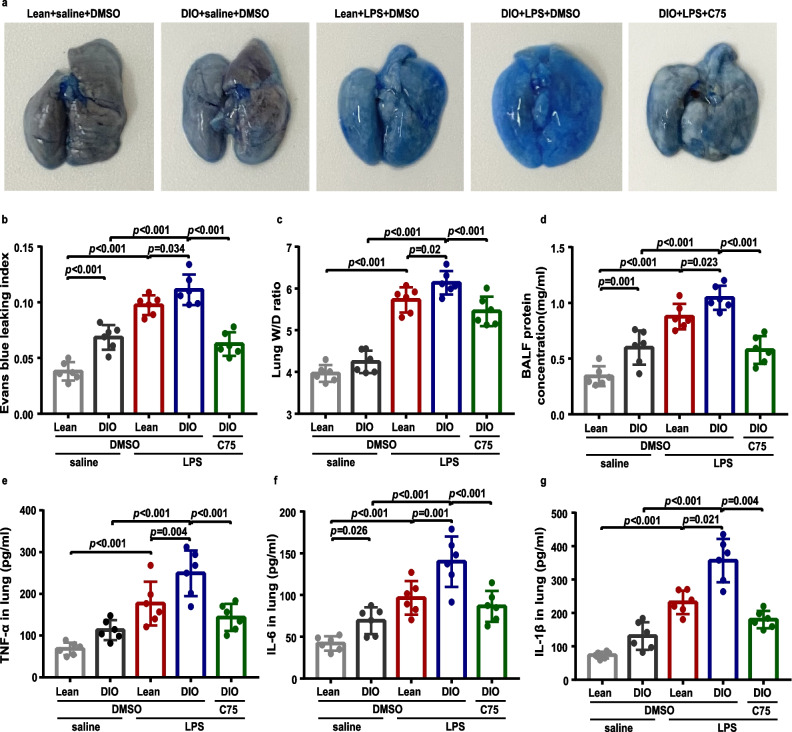
C75 significantly reduced lung vascular leakage and attenuated lung inflammation in DIO mice during LPS-induced ALI. The lean and DIO mice were intratracheally instilled LPS or saline for 8 h. C75 was intraperitoneally administered in DIO mice 30 min before LPS instillation. **a** Representative lung anatomy images following Evans blue dye injection into each group of mice. **b**–**d** Evans blue dye leaking index (**b**), lung wet/dry (W/D) ratios (**c**), and BALF protein concentration (**d**) in each group of mice were measured. **e–g** The levels of TNF-α (**e**), IL-6 (**f**), and IL-1β (**g**) in lung homogenates in each group were detected by ELISA assay. *n* = 6 mice in each group. One-way ANOVA and LSD or Games–Howell posthoc analysis. Data were represented as mean ± SD

To study the in vivo effect of FASN inhibition on endothelial barrier function in obesity, we next determined alterations in vascular permeability by EBD assay, lung wet-to-dry (W/D) ratio, and BALF protein concentration. The LPS challenge dramatically disrupted the lung endothelial barrier function, which was characterized by increased EBD contents in lung tissue (Fig. 10a, b), lung W/D ratio (Fig. 10c), and total protein levels in BALF (Fig. 10d). The total protein levels in the BALF and EBD contents in lung tissue were significantly elevated in obese mice compared with those in lean mice after 8 h of the LPS challenge (Fig. 10b, d), indicating that LPS-induced endothelial hyperpermeability was enhanced in obese mice. Furthermore, the lung W/D ratio, which was used as an indicator of pulmonary edema, was increased dramatically in obese mice compared with lean mice after 8 h of the LPS challenge (Fig. 10c). Additionally, obese mice exhibited much higher protein levels of TNF-α, IL-6, and IL-1β in the lung homogenates compared with lean mice after 8 h of the LPS challenge (Fig. 10e–g). However, the inhibition of FASN by C75 markedly attenuated lung endothelial barrier collapse in obese mice with LPS-induced ALI, which was determined by decreases in EBD contents in lungs (Fig. 10b), lung W/D ratio (Fig. 10c), total protein levels in BALF (Fig. 10d), and inflammatory cytokines in the lungs (Fig. 10e–g).

## Discussion

The present study revealed a previously unknown mechanism that FASN exerted deleterious effects on lung endothelial cells, which aggravated LPS-induced ALI under obesity. Meanwhile, our study provided evidence to show that FASN inhibition protected obese mice from aggravated ALI via amelioration of lung endothelial dysfunction.

Our in vivo experiments showed that the expression of FASN was augmented in lung endothelial cells from obese mice with LPS-induced ALI. Consistently, our further studies showed that the co-culture with adipocytes or treatment with PA, imitating metabolic stress in high fat diet-induced obesity, significantly elevated the expression levels of FASN in lung endothelial cells with or without LPS administration. Recent studies have shown that FASN, which initiated several biochemical and immunological pathways, was activated during acute inflammatory response [30–32]. Increased FASN gene expression in adipose tissue was positively correlated with the levels of circulating inflammatory cytokines IL-6 [12]. Additionally, the inhibition of FASN in the central nervous system protected high fat diet-induced obese mice from oxidative stress and systemic inflammation in response to endotoxin [33]. However, under the condition of obesity, whether and how FASN regulates lung inflammatory response during the development of LPS-induced ALI remains elusive. Previous study showed that the obesity subjects demonstrated a greater occurrence of pulmonary pathology changes compared with the control subjects [34]. We found that the inflammatory infiltration in lung pathological morphology and the protein levels in BALF from obese mice at baseline were increased, but other studies also found that there was no obvious lung pathological changes in obese mice at baseline [16, 35], which may be due to different obesity animal models or different feeding time of high-fat diet. We observed that representative histopathological alterations, such as thickened alveolar wall and inflammatory cell infiltration, and the levels of inflammatory cytokines overproduction were remarkably alleviated after the inhibition of FASN in the obese lungs with LPS-induced ALI. Meanwhile, we found that FASN inhibition with corresponding shRNAs or chemical inhibitor C75 significantly decreased the levels of LPS-induced inflammatory cytokines in MLECs under metabolic stress. Taken together, these results suggested that the expression levels of FASN in lung endothelial cells were augmented under obesity during LPS-induced ALI. FASN inhibition exerted a protective effect against lung injury in response to LPS in obesity.

In this study, we focused on investigating the mechanism by which FASN modulated lung endothelial homeostasis under obesity in LPS-induced ALI. It has been well documented that metabolic syndrome impaired vascular endothelial homeostasis [36, 37]. However, the effects of obesity on pulmonary vascular homeostasis and the underlying molecular mechanisms in ALI remain underexplored. Mitochondrial dysfunction impairs pulmonary microvascular barrier integrity and aggravates lung inflammatory injury following LPS stimulation [27, 28]. Previous studies showed that FASN was closely related to mitochondrial function [38–40]. Excessive production of mtROS, which contributes to the dissipation of MMP, is thought to be a crucial factor in the development of mitochondrial dysfunction [41]. Emerging evidence suggests that the suppression of FASN can reduce oxidative stress and increase mitochondrial membrane potential [38, 40]. In line with these findings, we observed that the inhibition of FASN significantly reversed the overproduction of mtROS and restored the mitochondrial membrane potential in MLECs under metabolic stress following LPS administration. We also found that FASN inhibition improved LPS-induced disorganized mitochondrial cristae ultrastructures in MLECs under metabolic stress. Our results also demonstrated that FASN inhibition restored the disruption of mitofusion protein Mfn2 and mitofission protein Drp1, suggesting a beneficial effect on the regulation of mitochondrial dynamics. Therefore, these results suggested that the FASN inhibition in obesity rescued mitochondrial dysfunction, thus contributing to the maintenance of lung endothelial homeostasis.

At present, little is known about how obesity affects the barrier function of the pulmonary vascular endothelium. Previous studies demonstrated that p38 MAPK signaling was activated and involved in chronic inflammation, insulin resistance, and proinflammatory responses to infection under obesity [42–44]. Meanwhile, NLRP3 exerts a predominant role in the pathogenesis of obesity-associated inflammatory diseases [45, 46]. A previous study reported that FASN regulated the transcription of NLRP3 through p38 MAPK signaling in macrophages [47]. Moreover, NLRP3 played a crucial functional role in disrupting vascular endothelial barrier function and inducing vascular endothelial hyperpermeability via inhibiting VE-cadherin, a core component of endothelial adherens junction protein [48, 49]. Similarly, in this study, we identified that the metabolic stress induced a further increase in the levels of phosphorylated p38 MAPK and NLRP3 and a further decrease in the levels of VE-cadherin in MLECs following LPS stimulation. Notably, aberrant upregulation of FASN in cancer cells reduced the expression of E-cadherin, which is a vital epithelial adherens junction protein [50, 51]. Strikingly, we noted that FASN inhibition or FASN deficiency in MLECs under metabolic stress remarkably increased VE-cadherin expression along with the downregulation of p38 MAPK phosphorylation and NLRP3 following LPS stimulation. These findings lead us to posit that FASN suppressed the expression of VE-cadherin via activating the p38 MAPK/NLRP3 signaling pathway in lung endothelial cells under metabolic stress in response to LPS. Endothelial permeability is normally determined by the level of adherens junction proteins that exerts a crucial role in preserving the endothelial barrier [52]. Meanwhile, we found that FASN inhibition promoted lung endothelial barrier repair under metabolic stress, as evidenced by the upregulation of VE-cadherin, increased TEER, and decreased permeability in MLECs following LPS stimulation. We further determined that FASN inhibition in obese mice dramatically restored the loss of VE-cadherin in lung endothelial cells and exerted a beneficial role in reducing lung vascular leakage during LPS-induced ALI. These results suggested that FASN inhibition increased VE-cadherin expression levels and thus contributed to improve endothelial barrier integrity and protecting against LPS-induced ALI under the condition of obesity.

There are some limitations of the current study. In our experiment, lean mice were fed with regular chow diet instead of ingredient matched diet, which may affect the experimental results due to different dietary components. C75 treatment is more complex than currently thought, and the effects require further clarification, especially with endothelial cells-specific FASN knockout mice in vivo experiments.

## Conclusions

Collectively, our findings revealed that upregulated FASN was involved in disrupting lung endothelial homeostasis, which probably led to enhanced susceptibility to LPS-induced ALI in obesity. FASN inhibition in obesity attenuated the exacerbation of lung inflammatory injury in response to LPS via rescuing lung endothelial dysfunction. Therefore, targeting FASN might be an impactful therapeutic approach to ameliorate LPS-induced ALI in obese individuals.

## Data Availability

The datasets used and/or analyzed during the current study available from the corresponding author on reasonable request.

## Abbreviations

FASN: Fatty acid synthase
ALI: Acute lung injury
LPS: Lipopolysaccharide
DIO: Diet-induced obesity
MLECs: Mouse lung endothelial cells
PA: Palmitic acid
MMP: Mitochondrial membrane potential
mtROS: Mitochondrial reactive oxygen species
p38 MAPK: P38 mitogen-activated protein kinase
TEER: Transendothelial electrical resistance
BALF: Bronchoalveolar lavage fluid
EBD: Evans blue dye
W/D: Wet-to-dry
IL-6: Interleukin-6
TNF-α: Tumor necrosis factor-alpha
IL-1β: Interleukin-1β
